# Identification and Functional Characterization of a Novel *SOX4* Mutation Predisposing to Coffin–Siris Syndromic Congenital Heart Disease

**DOI:** 10.3390/children12050608

**Published:** 2025-05-07

**Authors:** Zi Yan, Bin-Bin Dong, Yan-Jie Li, Chen-Xi Yang, Ying-Jia Xu, Ri-Tai Huang, Xing-Yuan Liu, Yi-Qing Yang

**Affiliations:** 1 Department of Pediatrics, Tongji Hospital, Tongji University School of Medicine, Shanghai 200065, China; yanzi@tongji.edu.cn; 2 Department of Pediatrics, Huashan Hospital, Fudan University, Shanghai 200040, China; dongbinbin@huashan.org.cn; 3 Department of Cardiology, Shanghai Chest Hospital, Shanghai Jiao Tong University School of Medicine, Shanghai 200030, China; lyj2014@alumni.sjtu.edu.cn; 4 Department of Cardiology, Shanghai Fifth People′s Hospital, Fudan University, Shanghai 200240, China; cxyang21@m.fudan.edu.cn (C.-X.Y.); xuyingjia@5thhospital.com (Y.-J.X.); yangyiqing@fudan.edu.cn (Y.-Q.Y.); 5 Department of Cardiovascular Surgery, Renji Hospital, Shanghai Jiao Tong University School of Medicine, Shanghai 200127, China; huangritai@renji.com; 6 Department of Cardiovascular Research Laboratory, Shanghai Fifth People’s Hospital, Fudan University, Shanghai 200240, China; 7 Department of Central Laboratory, Shanghai Fifth People’s Hospital, Fudan University, Shanghai 200240, China

**Keywords:** congenital heart disease, patent ductus arteriosus, transcription regulation, human molecular genetics, SOX4, biological assay

## Abstract

**Background/Objectives:** Congenital heart disease (CHD) occurs in ~1% of all live neonates globally, rendering it the most prevalent developmental anomaly affecting humans; this condition confers substantial infant morbidity and mortality worldwide. Although there is ample evidence to suggest a paramount genetic basis for CHD, the genetic etiologies underpinning the majority of CHD remain elusive. In the present study, *SOX4* was selected as a significant candidate gene for human CHD, mainly because SOX4 is abundantly expressed in both human and murine hearts during embryogenesis, and the knockout of *Sox4* in mice causes embryonic demise predominantly attributable to cardiovascular developmental defects. **Methods:** Sequencing analysis of *SOX4* was fulfilled in 248 probands affected with various types of CHD and the available relatives of the identified variation carrier as well as 262 unrelated healthy individuals. Functional analysis of the mutant SOX4 protein was conducted by utilizing a dual-reporter gene system. **Results:** a novel heterozygous *SOX4* variation, NM_003107.3:c.331G>T;p.(Glu111*), was discovered in a male proband with Coffin–Siris syndromic CHD. Genetic investigation of the proband’s available relatives revealed that the truncating variation co-segregated with the phenotype in the whole family. The nonsense variation was absent from 262 healthy controls. Functional analysis demonstrated that the Glu111*-mutant SOX4 lost transactivation on *NKX2.5* and *GATA4*, two well-established genes that are causative factors for CHD. Moreover, the Glu111* mutation nullified the synergistic transactivation between SOX4 and TBX20, another CHD-causing gene. **Conclusions:** These findings support *SOX4* as a causative gene accountable for familial Coffin–Siris syndromic CHD in humans. These findings may aid in developing personalized preventive and therapeutic strategies for patients with Coffin–Siris syndromic CHD.

## 1. Introduction

Congenital heart disease (CHD), which refers to the anomalous/incomplete development of the heart and valves, as well as endothoracic blood vessels during embryogenesis, occurs in ~1% of all living newborns and up to 10% of early miscarriages globally, rendering it the most prevalent form of human birth defect worldwide [1,2]. If minor cardiovascular developmental aberrations, such as a bicuspid aortic valve and patent foramen ovale are included, the overall incidence of CHD then rises to ~5% in live-born neonates [3]. As a condition affecting pediatric patients, CHD comprises an extensive assortment of cardiovascular malformations present at birth, encompassing patent ductus arteriosus (PDA), persistent truncus arteriosus (PTA), single ventricle (SV), interrupted aortic arch (IAA), aortic atresia (AA), ventricular septal defect (VSD), transposition of the great arteries (TGA), endocardial cushion defect (ECD), coarctation of the aorta (CoA), atrial septal defect (ASD), double outlet right ventricle (DORV), anomalous pulmonary venous connection (APVC), tetralogy of Fallot (TOF), aortic stenosis (AS), pulmonary valve stenosis (PVS), tricuspid atresia (TA), pulmonary atresia (PA), and left heart hypoplasia (LHH) [4]. Although minor simple cardiovascular developmental anomalies may spontaneously resolve or have no notable hemodynamic effect [1], severe complex cardiovascular developmental malformations may give rise to a degraded health-related quality of life [5], reduced physical exercise performance [6,7], malnutrition and poor weight gain [8], brain injuries and neurodevelopmental impairments [9,10], acute ischemic/thromboembolic stroke [11], abnormal lung development as well as pulmonary arterial hypertension [12,13], acute renal damage and chronic nephropathy [14], hepatic fibrosis and dysfunction [15], necrotizing enterocolitis [16], infective endocarditis [17], cardiac dysfunction and congestive/chronic heart failure [18,19], cardiac arrhythmias, including miscellaneous supraventricular and life-threatening ventricular dysrhythmias [20,21], and even premature cardiac demise [22,23]. Consequently, CHD is associated with a substantial increase in morbidity and mortality rates along with a socioeconomic burden on humans, highlighting the urgent need to ascertain the etiologies resulting in CHD [1].

The etiologies leading to CHD are multifactorial and complex, and both extrinsic/environmental pathogenic factors and intrinsic/genetic determinants have been demonstrated to play essential roles in the occurrence of CHD [24,25,26,27,28,29,30,31]. It has been reported that non-inheritable environmental risk factors may contribute to ~10% of CHD cases, although the precise mechanisms responsible for the development of CHD are largely unknown [32]. The well-known non-heritable risk factors for CHD encompass, but are not limited to, maternal illnesses (such as obesity, hypertension, mellitus diabetes, pre-eclampsia, malnutrition, and autoimmune disorder), infectious agents (such as influenza, rubella, and cytomegalovirus viruses), the consumption of tobacco, alcohol and medications, and a wide range of environmental exposures to toxic chemicals and air pollutants during the first trimester of pregnancy [32,33]. Furthermore, the ecological factors contributing to the development of CHD may also play a role in modifying the functional effects of genetic components underlying CHD, hence accounting for variabilities in the clinical manifestation of CHD [32]. However, ever-aggregating strong evidence consistently indicates that hereditary deficits play a major role in the occurrence of CHD [4,28,29,30,32], contributing to ~90% of CHD cases [32]. Currently, 20–30% of patients with CHD may be genetically diagnosed with identified CHD-causative heritable ingredients, including 3–25% copy number variations (deletion or duplication), 8–10% gross chromosomal abnormalities/aneuploidies, and 3–5% single gene mutations [34]. Notably, an ever-growing number of deleterious mutations in >100 genes have been reported to be accountable for a wide spectrum of CHD cases [29,30,31,32,34,35,36,37,38,39,40,41,42,43,44,45,46,47,48,49,50]. Thus far, the discovered genetic causes can be utilized to explain 30–45% of cases of CHD [32,34]. Nevertheless, owing to the pronounced genetic heterogeneity of CHD in the human population, a genetic diagnosis is still unresolved in up to 55% of CHD cases [34], which renders it reasonable to identify new genes contributing to CHD.

Recently, accumulating robust evidence suggests that sex-determining region Y (SRY)-related high-mobility group (HMG) box 4 (SOX4), a key member of the group C subfamily of a large family of so-called SOX transcription factors, exerts a crucial effect on the embryonic morphogenesis of multiple organs, where it is amply expressed and regulates the development of various tissues [51]. In mice, SOX4 is expressed predominantly at several sites (atrioventricular canal and outflow tract) of the heart during embryogenesis, and murine embryos with a targeted disruption of the *Sox4* gene have been shown to succumb to circulatory failure due to the impaired transformation of the endocardial ridges into the semilunar valves with no fusion of these ridges, as well as the defective outlet portion of the ventricular septum, an anomaly similar to the common arterial trunk (or transposition of the great arteries, or infundibular VSD in the least affected hearts) in humans [52,53]. In humans, *SOX4* mutations have been associated with Coffin–Siris syndrome (CSS), and approximately 50% of patients carrying pathogenic *SOX4* variations have congenital cardiovascular deformities, particularly VSD [51,54]; this indicates that human *SOX4* plays a key role in proper cardiovascular development, justifying *SOX4* as a preferable candidate gene for CHD in humans.

## 2. Materials and Methods

### 2.1. Ethical Approval and Informed Consent

The present patient–control study was performed in strict accordance with the ethical tenets proclaimed in the Declaration of Helsinki (revised in 2013). The protocol applied to human research was approved by the Ethics Committee of Tongji Hospital, Tongji University (approval code: LL(H)-09-07; approval date: 27 July 2009). The legal guardians of the research subjects signed the informed consent forms when recruiting the research subjects prior to sample collection. The identities of the research participants were encrypted and secured under the approved ethical regulations.

### 2.2. Human Research Participants

For the present human case–control study, 248 index patients who suffered from distinct forms of CHD were recruited. The available pedigree members of the probands with CHD were also enlisted. As the study controls, 262 unrelated non-CHD individuals were enrolled. A comprehensive clinical evaluation was performed for each research participant at the time of study entry, encompassing a thorough review of familial history and medical records, a detailed physical examination, echocardiographic images, and routine clinical laboratory test results. The clinical diagnosis and classification of CHD were made as previously described [3,40]. The inclusion criteria for the CHD case group were a definite diagnosis of CHD based on the color of Doppler echocardiograms or interventional/surgical proceedings and the provision of written informed consent. The exclusion criteria for the CHD case group were as follows: (i) there was a diagnosis of CHD, although there was a lack of objectively confirmed evidence, such as echocardiograms and medical records; (ii) there was a defined cause explainable for CHD; and (iii) there was no written informed consent. The inclusion criteria for the healthy control group were healthy individuals who were age- and sex-matched with the CHD group, had normal echocardiograms with no history of CHD, and had provided written informed consent. Eligible healthy individuals with a family history of CHD or with no written informed consent were excluded. Whole venous blood samples (1–3 mL) were collected from all study subjects and were kept immediately in a refrigerator at −80 °C until DNA extraction.

### 2.3. Genetic Investigation of Human SOX4

The purification of genomic DNA from the venous blood leucocytes of study participants was implemented by utilizing the Gentra Puregene Blood kit (cat. no. 158026; Qiagen, Inc., Hilden, Germany). Human genomic DNA was refrigerated at −20 °C prior to further use. The primers used to amplify the coding exon along with flanking partial untranslated regions of the human *SOX4* gene through polymerase chain reaction (PCR) were described elsewhere [55]. The actual primer pairs used for amplifying *SOX4* with only one exon were as follows: for exon 1 (part a) forward, 5′-CTCTCTTTACCCACCTCCGC-3′ and reverse, 5′-GACCTTGTCTCCCTTCTCCC-3′ (amplicon size, 643 bp); for exon 1 (part b) forward, 5′-GCCCAGGAAGAAGGTGAAGT-3′, and reverse, 5′-GCGCCCTCCTCCTCGTACAG-3′ (amplicon size, 603 bp); for exon 1 (part c) forward, 5′-TGGCGGAGAAGAAGGTGAAG-3′, and reverse, 5′-TCGTCTGTCCTTTTCGTTTCT-3′ (amplicon size, 647 bp). With the *SOX4*-specific primers and the Phusion^™^ Plus DNA Polymerase (cat. no. F630L; Thermo Fisher Scientific, Inc., Waltham, MA, USA), the amplification of *SOX4* from the genomic DNA of each study subject via PCR was completed under the MiniAmp^™^ Plus Thermal Cycler (Applied Biosystems; Thermo Fisher Scientific, Inc.), as per the manufacturer’s instructions. The amplicons were fragmented via electrophoresis on a 1.6% agarose gel and isolated utilizing the GeneJET Gel Extraction kit (cat. no. K0691; Thermo Fisher Scientific, Inc.). With the purified amplicon as a template, a sequencing PCR was carried out using the BigDye^®^ Terminator v3.1 Cycle Sequencing kit (cat. no. 4337456; Applied Biosystems; Thermo Fisher Scientific, Inc.) and a forward or backward *SOX4*-specific primer under the T100 Thermal Cycler (Bio-Rad Laboratories, Inc., Hercules, CA, USA) as per the manufacturer’s operating instructions. The actual primers used for sequencing SOX4 were the same as the actual primers used for amplifying SOX4. The sequencing PCR products were isolated using the BigDye^®^ XTerminator^™^ Purification kit (cat. no. 4376487; Applied Biosystems; Thermo Fisher Scientific, Inc.) and sequenced by capillary electrophoresis with the 3730 DNA Analyzer (Applied Biosystems; Thermo Fisher Scientific, Inc.) as per the manufacturer’s protocols. For an identified *SOX4* variation, the gnomAD database (https://gnomad.broadinstitute.org/gene/ENSG00000124766?dataset=gnomad_r4; accessed on 13 November 2024) and dbSNP database (https://www.ncbi.nlm.nih.gov/snp/?term=SOX4; accessed on 13 November 2024) were retrieved to ascertain its novelty.

### 2.4. Preparation of Gene Expression Constructs

As described elsewhere [55], the SOX4-pCI vector expressing wild-type human SOX4 was generated. The Glu111*-mutant SOX4-pCI construct was produced through PCR-based site-directed mutagenesis by utilizing the Phusion^™^ Site-Directed Mutagenesis Kit (cat. no. F541; Thermo Fisher Scientific, Inc.) and a complimentary pair of primers carrying the identified *SOX4* mutation (forward, 5′-ATCCCTTTCATTCGATAGGCGGAGCGGCTGC-3′, and reverse, 5′-GCAGCCGCTCCGCCTATCGAATGAAAGGGAT-3′) and was verified via direct sequencing assay. The human TBX20-pcDNA3.1 vector was created as described elsewhere [56]. The nucleotide accession number to the cDNA of human *TBX20* used in the pcDNA3.1 vector was NM_001077653.2. With human genomic DNA used as a template, a 1621-bp DNA segment (from −314 to −1934, with the transcriptional initial nucleotide numbered as +1) of the human *NKX2.5* gene (GenBank accession no. NC_000005.10) was PCR-amplified with a specific pair of primers (forward, 5′-GAAGAGCTCGCATCCTCCACAGACTAGAC-3′, and reverse, 5′-GAAAGATCTGGCAGGGCTGGATGGGTGTG-3′). The produced *NKX2.5* promoter was digested with *Sac*I (cat. no. ER1132; Thermo Fisher Scientific, Inc.) and *Bgl*II (cat. no. ER0082; Thermo Fisher Scientific, Inc.), and subsequently inserted at the *Sac*I-*Bgl*II sites into the pGL3-Basic plasmid (cat. no. E1751; Promega Corporation, Madison, WI, USA) to yield the *NKX2.5* promoter-induced firefly luciferase expression plasmid (NKX2.5-luc). Similarly, a 1913-bp DNA segment (from −13,448 to −15,360) of the human *GATA4* gene (GenBank accession no. NC_000008.11) was PCR-amplified with a specific pair of primers (forward, 5′-CAAGAGCTCTGGTGGAGAGATGGGTGAG-3′, and reverse, 5′-CAACTCGAGGAGTACAGGTGCGCACCAC-3′). The resultant *GATA4* promoter was doubly cut with the restriction endonucleases of *Sac*I (cat. no. ER1132; Thermo Fisher Scientific, Inc.) and *Xho*I (cat. no. ER0692; Thermo Fisher Scientific, Inc.), and subsequently subcloned at the *Sac*I-*Xho*I sites into the pGL3-Basic plasmid (cat. no. E1751; Promega Corporation) to generate the *GATA4* promoter-driven firefly luciferase reporter vector (GATA4-luc).

### 2.5. Functional Characterization of Glu111*-Mutant SOX4 with Dual Report Genes

As described elsewhere [55,57], HeLa (cat. no. SCSP-504; National Collection of Authenticated Cell Cultures, Shanghai, China) and 293 cells (cat. no. SCSP-5209; National Collection of Authenticated Cell Cultures, China) were cultured and transfected with appropriate expression vectors with the Lipofectamine^™^ 3000 Transfection Reagent (cat. no. L3000075; Invitrogen; Thermo Fisher Scientific, Inc.). In brief, the HeLa cells were transiently transfected with 400 ng of empty pCI vector (cat. no. E1731; Promega Corporation), 400 ng wild-type SOX4-pCI vector, 400 ng Glu111*-mutant SOX4-pCI vector, 200 ng wild-type SOX4-pCI vector + 200 ng empty pCI vector, or 200 ng wild-type SOX4-pCI vector + 200 ng Glu111*-mutant SOX4-pCI vector, in combination with 1.2 μg NKX2.5-luc and 4 ng pGL4.75 (cat. no. E6931; Promega Corporation). For the synergistic transactivation measurement, 293 cells were transiently transfected with 1.0 μg GATA4-luc and 2 ng pGL4.75 (cat. no. E6931; Promega Corporation) together with 400 ng of each expression vector (empty pCI, wild-type SOX4-pCI, Glu111*-mutant SOX4-pCI, and wild-type TBX20-pcDNA3.1, separately or in combination). In this case, the empty vector pCI (cat. no. E1731; Promega Corporation) was utilized as an external negative control, and the *Renilla* luciferase expression vector pGL4.75 (cat. no. E6931; Promega Corporation) was employed as an internal control reporter to balance transfection efficiency. Cellular transfection experiments were performed in three independent replicates of each expression vector. The cells were harvested at 48 h post cellular transfection and then lysed. As previously described [55], the cellular lysates were utilized to quantitatively measure the dual luciferase activities on the GloMax^®^ Discover System (Promega Corporation) with the Dual-Glo^®^ Luciferase Assay System (cat. no. E2920; Promega Corporation).

### 2.6. Statistics

The results are presented as the arithmetic mean ± standard deviation (SD) for continuous parameters or frequency number (percentage) for categorical variables. The quantitative data (such as age and the *NKX2.5* or *GATA4* promoter activity) were compared utilizing the Student’s *t*-test between two groups or one-way analysis of variance (ANOVA) followed by Tukey’s post hoc test among three or more groups. The comparisons of categorical variables (such as sex and a positive family history of CHD) between two groups were implemented utilizing Pearson’s chi-squared test or Fisher’s exact test when indicated. Statistical analysis was completed employing SPSS 17.0 (IBM Corp., Armonk, NY, USA), and two-tailed tests were applied universally. A value of *p* < 0.05 was considered to indicate a statistically significant difference.

## 3. Results

### 3.1. Baseline Clinical Data of the Research Participants

In the present patient–control study, 248 index patients affected with CHD (117 female probands and 131 male probands, with a mean age of 3.86 ± 2.51 years) were clinically explored in contrast to 262 unrelated healthy subjects (125 female healthy subjects and 137 male healthy subjects, with an average age of 3.91 ± 2.37 years). The study participants were all enrolled from the Chinese population of the Han race in Shanghai, China. All the patients had definite echocardiograms documenting CHD, while the echocardiograms of the control subjects were normal, with no evidence indicating cardiovascular developmental aberrations. Among the 248 probands with different forms of CHD, 51 (20.56%) probands had a positive familial history of CHD, whereas all 262 control subjects had no familial history of CHD. No included research participants had known pathogenic factors responsible for CHD, and the majority of patients with CHD underwent cardiovascular surgery or catheter-based cardiac interventional therapy. The demographic and baseline phenotypic features of the 248 probands suffering from CHD are provided in Table 1.

### 3.2. Discovery of a New SOX4 Mutation Accountable for CHD

**Figure 1:**
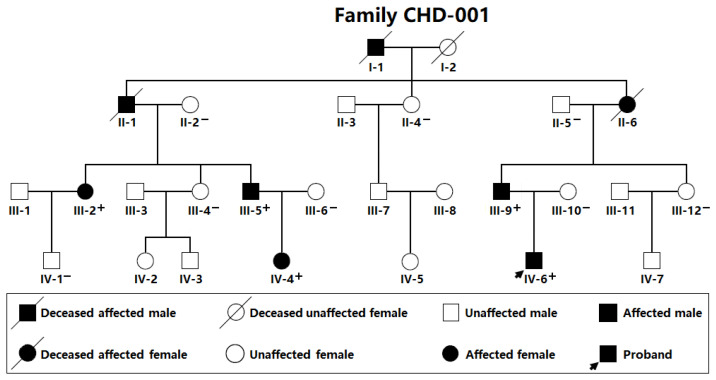
Pedigree of the family with congenital heart disease. The family was arbitrarily designated as Family CHD-001. Roman–Arabic numerals were used to identify family members. CHD: congenital heart disease; +: a carrier of the identified heterozygous SOX4 mutation (c.331G>T, equal to p.Glu111*); and −: a non-carrier.

By the sequencing assay of the *SOX4* gene in 248 probands with a wide variety of CHD, a novel *SOX4* mutation in a heterozygous status, NM_003107.3:c.331G>T; p.(Glu111*), was discovered in a male proband with PDA. No *SOX4* mutation was discovered in the other 247 probands with a wide variety of CHD. A genetic examination of the available relatives of the mutation carrier revealed that the mutation co-segregated with PDA in the whole family, which was arbitrarily designated as Family CHD-001, as illustrated in Figure 1.

**Figure 2:**
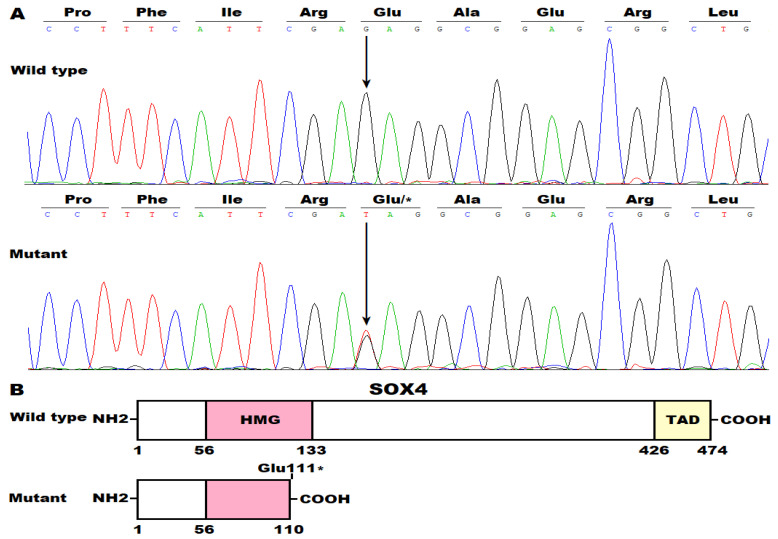
Representative *SOX4* sequence chromatograms from the family members and schemas of structural domains of SOX4. (**A**) Sequence chromatograms displaying the heterozygous *SOX4* mutation (c.331G>T) identified in a family member with patent ductus arteriosus (mutant) along with the wild-type control (G/G) in a healthy individual (wild-type). (**B**) Schemas delineating the critical structural domains of wild-type SOX4 and its mutant with the Glu111* mutation noted. COOH, carboxyl-terminus; TAD, transactivation domain; HMG, high-mobility group; and NH2, amino-terminus. Here a blue color line indicates cytosine (C), a red color line indicates thymine (T), a green color line indicates adenine (A), and a black color line indicates guanine (G).

Moreover, a sequencing assay of *SOX4* was completed in 262 control subjects; no mutation was detected. The identified *SOX4* mutation was not found in either the gnomAD database (logged in on 13 November 2024) or the dbSNP database (accessed on 13 November 2024), confirming the novelty of the observed *SOX4* variation causative for CHD. Notably, all five living PDA-affected members (III-2, III-5, III-9, IV-4, and IV-6 from Family CHD-001) of the mutation-carrying proband (IV-6 from Family CHD-001) underwent catheter-based interventional treatment for PDA. In addition, two patients with PDA (III-5 and IV-4 from Family CHD-001) also had VSD, which was to be surgically closed. The grandmother of the proband (II-6 from Family CHD-001) succumbed due to sudden cardiac death at the age of 51 years. In addition to CHD, all the patients also suffered from congenital craniofacial dysmorphisms and hypoplasia of the fifth distal phalanges/nails, as well as mild intellectual disability, so-called Coffin–Siris syndrome (CSS). The representative sequencing chromatograms of family members’ *SOX4* are provided in Figure 2A. The structural domains of the human wild-type SOX4 and Glu111*-mutant SOX4 are delineated in Figure 2B.

The clinical cardiovascular profile of the living patients with CHD from Family CHD-001 is presented in Table 2.

### 3.3. Failure of Glu111*-Mutant SOX4 to Induce NKX2.5 Expression

**Figure 3:**
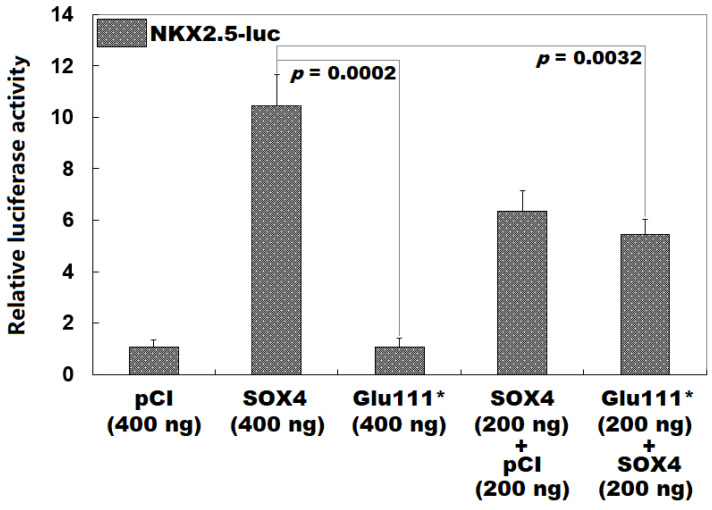
No transactivation of *NKX2.5* by Glu111*-mutant SOX4. In cultured HeLa cells transfected with the appropriate expression plasmids, dual-reporter gene gauges of the *NKX2.5* promoter activity revealed that the Glu111*-mutant SOX4 lost transactivation of *NKX2.5*. For each gene expression construct, three independent cellular transfection experiments were executed.

As illustrated in Figure 3, in the cultured HeLa cells expressing required expression vectors, the wild-type SOX4-pCI vector (SOX4) and Glu111*-mutant SOX4-pCI vector (Glu111*) transactivated *NKX2.5* by ~10-fold and ~1-fold, respectively (SOX4 vs. Glu111*: *t* = 12.7981; *p* = 0.0002). When SOX4 was co-expressed with Glu111*, the induced transactivation of *NKX2.5* was ~5-fold, while when SOX4 was co-expressed with the empty pCI vector (pCI), the induced transactivation of *NKX2.5* was ~6-fold (SOX4 vs. SOX4 + Glu111*: *t* = 6.3387; *p* = 0.0032; SOX4 + pCI vs. SOX4 + Glu111*: *t* = 1.5460; *p* = 0.1970). Equivalent results were acquired when a comparison was conducted among all five groups (*F* = 86.633, *p* = 1.017 × 10^−7^) as follows: pCI vs. SOX4, *t* = 9.3767; *p* < 0.0001; pCI vs. Glu111*, *t* = 0.0033; *p* > 0.9999; pCI vs. SOX4 + pCI, *t* = 5.2767; *p* < 0.0001; pCI vs. SOX4 + Glu111*, *t* = 4.3767; *p* = 0.0002; SOX4 vs. Glu111*, *t* = 9.3733; *p* < 0.0001; SOX4 vs. SOX4 + pCI, *t* = 4.1000; *p* = 0.0003; SOX4 vs. SOX4 + Glu111*, *t* = 5.0000; *p* < 0.0001; Glu111* vs. SOX4 + pCI, *t* = 5.2733; *p* < 0.0001; Glu111* vs. Glu111* + SOX4, *t* = 4.3733; *p* = 0.0002; and SOX4 + pCI vs. Glu111* + SOX4, *t* = 0.9000; *p* = 0.5852.

### 3.4. No Synergistic Activation of GATA4 by Glu111*-Mutant SOX4 with TBX20

**Figure 4:**
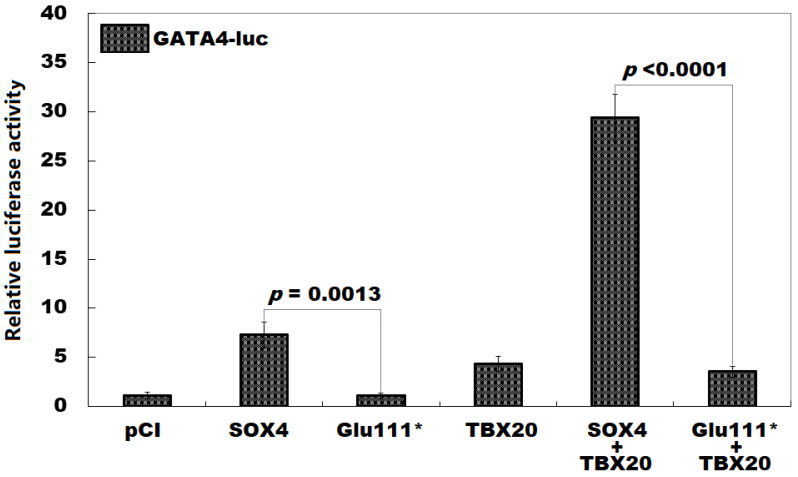
No synergistic transactivation between Glu111*-mutant SOX4 and TBX20. In cultured 293 cells transfected with the appropriate expression plasmids, dual-luciferase examinations of the *GATA4* promoter activity unveiled that the Glu111*-mutant SOX4 lost the ability to transactivate *GATA4* alone or in synergy with TBX20. For each gene expression plasmid, three independent cellular transfection experiments were fulfilled.

As suggested in Figure 4, in cultured 293 cells expressing required recombinant vectors, SOX4 and Glu111* transactivated *GATA4* by ~7-fold and ~1-fold, respectively (SOX4 vs. Glu111*, *t* = 7.9995; *p* = 0.0013). In co-expression with TBX20, SOX4 and Glu111* transactivated *GATA4* by ~32-fold and ~11-fold, respectively (SOX4 + TBX20 vs. Glu111* + TBX20, *t* = 18.2716; *p* < 0.0001). Equal results were obtained when a comparison was implemented among all six groups (*F* = 247.530, *p* = 1.136 × 10^−11^) as follows: pCI vs. SOX4, *t* = 6.2000; *p* = 0.0004; pCI vs. Glu111*, *t* = 0.0133; *p* = 1.0000; pCI vs. TBX20, *t* = 3.2667; *p* = 0.0503; pCI vs. SOX4 + TBX20, *t* = 28.3000; *p* < 0.0001; pCI vs. Glu111* + TBX20, *t* = 2.4500; *p* = 0.1937; SOX4 vs. Glu111*, *t* = 6.2133; *p* = 0.0004; SOX4 vs. TBX20, *t* = 2.9333; *p* = 0.0887; SOX4 vs. SOX4 + TBX20, *t* = 22.1000; *p* < 0.0001; SOX4 vs. Glu111* + TBX20, *t* = 3.7500; *p* = 0.0218; Glu111* vs. TBX20, *t* = 3.2800; *p* = 0.0492; Glu111* vs. SOX4 + TBX20, *t* = 28.3133; *p* < 0.0001; Glu111* vs. Glu111* + TBX20, *t* = 2.4633; *p* = 0.1897; TBX20 vs. SOX4 + TBX20, *t* = 25.0333; *p* < 0.0001; TBX20 vs. Glu111* + TBX20, *t* = 0.8167; *p* = 0.9541; and SOX4 + TBX20 vs. Glu111* + TBX20, *t* = 25.8500; *p* < 0.0001.

## 4. Discussion

In the present human case–control study, a novel *SOX4* mutation, NM_003107.3:c.331G>T;p.(Glu111*), was discovered in a family with CHD, which co-segregated with CHD in the entire family. The mutant allele was not discovered in either of the 262 control subjects, and it was also not found in the gnomAD and dbSNP databases. The biological exploration demonstrated that Glu111*-mutant SOX4 lost transactivation of the target genes of *NKX2.5* and *GATA4*, two well-established CHD-causing genes [58,59,60,61]. Moreover, the Glu111* mutation nullified the synergic transactivation between SOX4 and TBX20, another well-established CHD-causing gene [62,63]. These results convincingly indicate that dysfunctional *SOX4* contributes to the molecular pathogenesis of CHD in the mutation carriers and may help to provide *SOX4* gene-related diagnosis and treatment strategies for CHD patients harboring *SOX4* mutations.

In humans, *SOX4* is located on chromosome 6p22.3, encoding a transcription factor protein with 474 amino acids [55]. Previous investigations indicated that SOX4 is widely expressed in the cardiovascular systems of mice and humans, playing a pivotal role in proper embryogenesis and the structural remodeling of the hearts and vessels after birth [51,52,53,55,64,65]. Of note, SOX4 has been experimentally substantiated to mediate the expression of Cx43, separately or in synergy with the T-box family of transcription factors, encompassing TBX2, TBX3, and TBX5 [66]. Furthermore, the *GJA1*, *TBX2*, *TBX3*, and *TBX5* genes all play key roles in cardiovascular development, and deleterious mutations in these genes have been found to cause CHD [67,68,69,70,71,72]. In the present human study, a novel *SOX4* mutation was uncovered to co-segregate with CHD in a family with CHD, and the functional decipher revealed that the mutant SOX4 failed to transactivate its two representative target genes of *NKX2.5* and *GATA4*, alone or synergistically with TBX20. These observational results suggest *SOX4* haploinsufficiency as an alternative molecular pathogenesis of human CHD.

Strong evidence from animal experiments demonstrates that genetically defective *SOX4* leads to CHD [52,53,73]. In mice, SOX4 has been demonstrated to be predominantly expressed at the atrioventricular canal and outflow tract of the heart during embryogenesis, and murine embryos with a targeted deletion of the *Sox4* gene were shown to not survive on embryonic day 14 due to circulatory failure caused by severe malformation of the cardiac outflow tract [52,53]. These *Sox4*-null embryos manifested no other apparent malformations than cardiovascular defects, including hypoplastic endocardial cushions leading to incomplete ventricular septation and fusion of the aortic and pulmonary trunks, an abnormality such as a common arterial trunk, a transposition of the great arteries, or an infundibular VSD in the least affected hearts in humans, and hypoplastic semilunar valves, resulting in arterial blood backflow [52,53]. Moreover, conditional *Sox4*-null mice (*Sox4*^fl-/fl-^) died from the same congenital cardiovascular malformations as found in *Sox4*^−/−^ mice [73]. Additionally, though *Sox11*-null mice died shortly after birth, due to cardiac outflow tract deformations encompassing the common arterial trunk and the double outlet of the right ventricle and membranous VSD, the additional knockout of *Sox4* aggravated the phenotype generated by the disruption of *Sox11*, resulting in an increased incidence of outflow tract defects from 8 to 100% [74]. Therefore, these results from animal experiments suggest that *SOX4* is a crucial player in regulating cardiovascular development, and pathogenic *SOX4* mutation causes predisposition to the development of CHD in humans.

**Figure 5:**
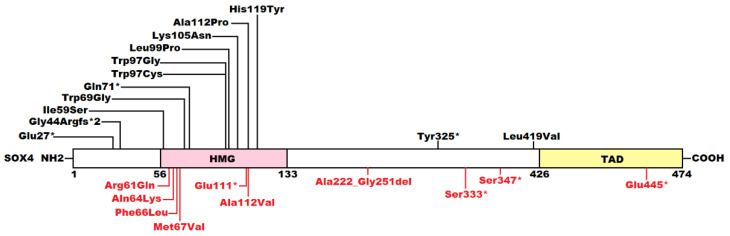
Schematic diagram of SOX4 protein showing pathogenic variants responsible for Coffin–Siris syndrome. The SOX4 variants reported to cause Coffin–Siris syndrome without cardiovascular developmental deformities are displayed above the protein, while the SOX4 variants reported to cause Coffin–Siris syndrome with cardiovascular developmental malformations are shown below in red. COOH, carboxyl-terminus; TAD, transactivation domain; HMG, high-mobility group; and NH2, amino-terminus. Here * means a stop codon.

In humans, multiple malicious mutations in *SOX4* have been reported to cause CSS, and about 50% of patients with CSS harboring pathogenic *SOX4* mutations have CHD, particularly VSD [51,54,75,76,77]. CSS is classically characterized by the hypoplasia/aplasia of the distal fifth phalanx or nail and additional digits, dysmorphic facial features, developmental/cognitive delays, hypotonia, and congenital organ system aberrations, including malformations of the heart, gastrointestinal tract, genitourinary tract, and nervous systems [51,54,75,76,77]. Other clinical manifestations of CSS include feeding difficulties, hypertrichosis, sparse scalp hair, hearing impairment, and ophthalmologic anomalies [51,54,75,76,77]. Recently, atrial fibrillation was reported to be a major clinical manifestation of CSS caused by a novel *SOX4* mutation [55]. In the present study, CHD was found to be a major clinical manifestation of CSS resulting from a novel *SOX4* loss-of-function mutation, thereby underscoring the critical roles of *SOX4* in human cardiovascular development. As listed in Figure 5, to date, at least 23 pathogenic missense, nonsense, and frameshift variants in *SOX4* have been reported to cause CSS globally (encompassing one nonsense *SOX4* variant identified in the present study), of which 10 pathogenic *SOX4* variants have been linked to various cardiovascular developmental abnormalities, including VSD, ASD, double aortic arch, abnormal pulmonary valve, anomalous coronary artery, and aortic aneurism [51,54,75,76,77].

In conclusion, the present study first indicates that genetically defective *SOX4* contributes to familial Coffin–Siris syndromic CHD, suggesting that CHD may be a major clinical manifestation of CSS caused by *SOX4* mutation in a minority of patients. The data presented herein may provide potential personalized preventive and therapeutic strategies for Coffin–Siris syndromic CHD caused by *SOX4* mutations.

## Figures and Tables

**Table 1:** Demographic and baseline phenotypic features of the patient cohort comprising 248 probands suffering from an extensive assortment of congenital heart disease.

Variables	Number or Mean	Percentage or Range
Demographic characteristics		
Male probands	131	52.82
Female probands	117	47.18
Age at the time of enrollment (year)	3.86 ± 2.51	0.25–7.17
A positive family history of CHD	51	20.56
Distribution of various forms of CHD		
VSD	63	25.40
ASD	56	22.58
PDA	38	15.32
DORV	12	4.84
TOF	10	4.03
PTA	5	2.02
TGA	4	1.61
HLV	2	0.81
AS	2	0.81
PS	1	0.40
APVC	1	0.40
SV	1	0.40
PCAC	1	0.40
VSD + PDA	16	6.46
VSD + ASD	12	4.84
DORV + VSD	8	3.23
ASD + PDA	6	2.42
TGA + VSD	5	2.02
TOF + ASD	3	1.21
PTA + VSD	2	0.81
Incidence of cardiac dysrhythmias		
AVB	12	4.84
AF	5	2.02
Medical management		
Catheter-based therapy	139	56.05
Surgical treatment	87	35.08
Follow-up observation	22	8.87

CHD: congenital heart disease; VSD: ventricular septal defect; ASD: atrial septal defect; PDA: patent ductus arteriosus; DORV: double outlet right ventricle; TOF: tetralogy of Fallot; PTA: persistent truncus arteriosus; TGA: transposition of the great arteries; HLV: hypoplastic left ventricle; AS: aortic stenosis; PS: pulmonary stenosis; APVC: anomalous pulmonary venous connection; SV: single ventricle; PCAC: partial common atrioventricular canal; AVB: atrioventricular block; and AF: atrial fibrillation.

**Table 2:** Clinical cardiovascular characteristics of the living patients from Family CHD-001 suffering from congenital heart disease.

Individual (Family CHD-001)	Sex	Age (Years)	Cardiovascular Structural Malformations
III-2	Female	31	PDA
III-5	Male	27	PDA, VSD
III-9	Male	29	PDA
IV-4	Female	2	PDA, VSD
IV-6	Male	4	PDA

CHD: congenital heart disease; PDA: patent ductus arteriosus; and VSD: ventricular septal defect.

## Data Availability

The data supporting the findings of this investigation are available upon a reasonable request. The data are not publicly available due to ethical requirement.
